# A Gene-Phenotype Network for the Laboratory Mouse and Its Implications for Systematic Phenotyping

**DOI:** 10.1371/journal.pone.0019693

**Published:** 2011-05-19

**Authors:** Octavio Espinosa, John M. Hancock

**Affiliations:** 1 Bioinformatics Group, MRC Mammalian Genetics Unit, Harwell, Oxfordshire, United Kingdom; 2 Department of Biochemistry, University of Oxford, Oxford, United Kingdom; Centre for Genomic Regulation, Spain

## Abstract

The laboratory mouse is the pre-eminent model organism for the dissection of human disease pathways. With the advent of a comprehensive panel of gene knockouts, projects to characterise the phenotypes of all knockout lines are being initiated. The range of genotype-phenotype associations can be represented using the Mammalian Phenotype ontology. Using publicly available data annotated with this ontology we have constructed gene and phenotype networks representing these associations. These networks show a scale-free, hierarchical and modular character and community structure. They also exhibit enrichment for gene coexpression, protein-protein interactions and Gene Ontology annotation similarity. Close association between gene communities and some high-level ontology terms suggests that systematic phenotyping can provide a direct insight into underlying pathways. However some phenotypes are distributed more diffusely across gene networks, likely reflecting the pleiotropic roles of many genes. Phenotype communities show a many-to-many relationship to human disease communities, but stronger overlap at more granular levels of description. This may suggest that systematic phenotyping projects should aim for high granularity annotations to maximise their relevance to human disease.

## Introduction

With the advent of well-annotated genome sequences for humans and other mammals the focus of genetic analysis is turning to the relationship between phenotype and genotype. The laboratory mouse is central to this analysis because of its ease of genetic manipulation and short generation time [1]. Mouse genetics has a history of over 100 years and has accumulated a large repository of information on mouse genes and the phenotypes associated with them [2]. As a model organism, the laboratory mouse benefits from a comprehensive set of gene function information that it is not possible to obtain in humans and therefore represents a unique opportunity for more comprehensive analyses in a mammal. However gaining an overview of gene-phenotype relationships is difficult using individual database searches [3]. There is therefore a need to develop a structure within which these relationships can be investigated in an integrated way.

Phenotype description in the mouse differs from that in humans in focusing on individual observable phenotypes rather than diseases, which are less clear-cut entities [4], [5]. A major strength of mouse phenotype data is the common use of a well-developed ontology to describe abnormal mouse phenotypes, the Mammalian Phenotype ontology (MP) [6]. This ontology, which is made up of 10,751 terms and 16 levels (as of 12.11.2010), contains most of the terms used by mouse geneticists to characterize abnormal mouse phenotypes. There are currently two major repositories for mouse phenotype information which hold phenotype information on genes annotated with MP: the Mouse Genome Database [2], which collects manually curated annotations for mouse genes, and EuroPhenome [7], which is a repository of the results of high-throughput phenotyping screens. A natural way to integrate phenotype and genotype data is to construct a bipartite graphical structure consisting of genes and terms from the MP, thereby linking MP terms by shared genes and genes by shared MP terms, in a manner analogous to the gene-disease network structure developed by Goh et al [8] for humans.

Goh et al [8] identified a number of significant features in their disease-gene network, in particular a tendency for genes associated with similar disorders to interact at the protein level and to show coexpression. Following on from this analysis, much emphasis has been placed on the prediction of gene function (normally in the form of Gene Ontology (GO) [9] terms), particularly in the context of genome-wide association studies. Butte and Kohane [10] related differentially expressed genes to disease, phenotype, experimental context and environment terms contained within gene annotations, emphasising the use of MeSH (Medical Subject Heading) terms to structure unstructured textual annotations. Disease-gene networks have also been applied to the problem of prioritising potential disease genes [11], [12], [13]. More broadly, Marcotte and co-workers have developed genome-wide functional gene networks using data from a variety of species to predict genes associated with particular phenotypes [14], [15], [16].

Network approaches have also been applied in the mouse. A systematic assessment of methods for predicting mouse gene function has shown the value of network-based approach in gene function prediction [17], [18]. Guan et al [19] generated a Bayesian network linking PPI, phenotype, disease, gene expression and phylogenetic profiles with the aim of functionally linking mouse genes and predicting gene function and pathway components. They found a strong dependence between MP phenotype terms and disease terms and so combined them for use in function prediction. In an alternative approach to cross-species linking of phenotype terms, Groth et al developed a text-, rather than ontology-based approach [20].

Network structures have a range of possible applications beyond the prediction of gene function. Whereas gene function prediction is important for the molecular dissection of phenotype and disease, the initial identification of disease models in the mouse (and other model organisms) usually starts by the identification of phenotypes in the model organism which mirror phenotypes associated with diseases in humans. These are then used as an access point to the genes and pathways involved in these pathways (forward genetics). It is therefore important to develop a formal link between phenotype ontologies and genes in order to characterise the relationship between phenotypes and their underlying genetics, and to allow best use to be made of gene annotations arising from projects, such as EUMODIC (http://www.eumodic.org/), which aim to systematically phenotype knockouts of every gene in the mouse genome. In this paper we explore the direct linking of MP terms to genes. Here we describe a gene-phenotype network constructed defined using mouse data from the MGD and Europhenome databases which links genes by shared MP terms and MP terms by shared genes. We compare the structure of this network with the human disease network and evaluate its utility for phenotype prediction and analysis and ontology refinement.

## Results

### Annotation granularity in the MP Ontology and GO

Unlike the construction of disease-gene bipartite networks [8], which simply involves linking gene names or identifiers with disease names, construction of a mouse gene-phenotype network requires the linking of MP ontology terms to gene names and IDs. The MP ontology (like other ontologies including the GO [9]) is structured such that terms have relationships between them so as to form a directed acyclic graph (DAG), allowing for varying levels of descriptive granularity. The ontologies are therefore organised into levels with different degrees of granularity. This introduces an additional level of complexity into the analysis. We therefore firstly considered the use of the different granularity levels of MP and GO ontology terms for annotation in two primary data sources.

The level immediately nearest the root of an ontology's DAG is termed level 1. This is the highest level and contains the most broad, generally descriptive (least granular) terms in the ontology. Specific (more granular) terms are labelled as lying at increasing level numbers corresponding to the number of nodes lying above them in the ontology's hierarchy. MP (and GO) annotations for mouse employ mixed levels of ontology terms. We therefore characterised the most frequent level of annotation used. To do this for MP terms we used MGD, which contains the largest public domain corpus of MP annotations. The results are presented in **Figure S1**. The MP ontology showed a single peak of most frequent annotation at level 5. We therefore focussed on MP level 5 in the subsequent analysis and we also considered MP level 8, after which annotation usage declines sharply, to characterise the effect of using a more granular level of annotation on the overall results. These levels also provided a compromise between granularity and retention of annotations. To inform analysis of GO terms associated with genes, we also considered the levels of the three GO sub-ontologies used in gene annotation in Ensembl. The “biological process” (BP) of the GO also showed a single peak of most frequent annotation at level 5. However the “cellular component” (CC) of GO showed two peaks at levels 2 and 5 while the “molecular function” (MF) of GO has three peaks at levels 2, 4, and 6. We therefore used level 5 of the BP and CC branches of the GO and level 2 of the MF branch to investigate functional enrichment of gene communities.

### Characterisation of the mouse gene-phenotype network

**Figure 1:**
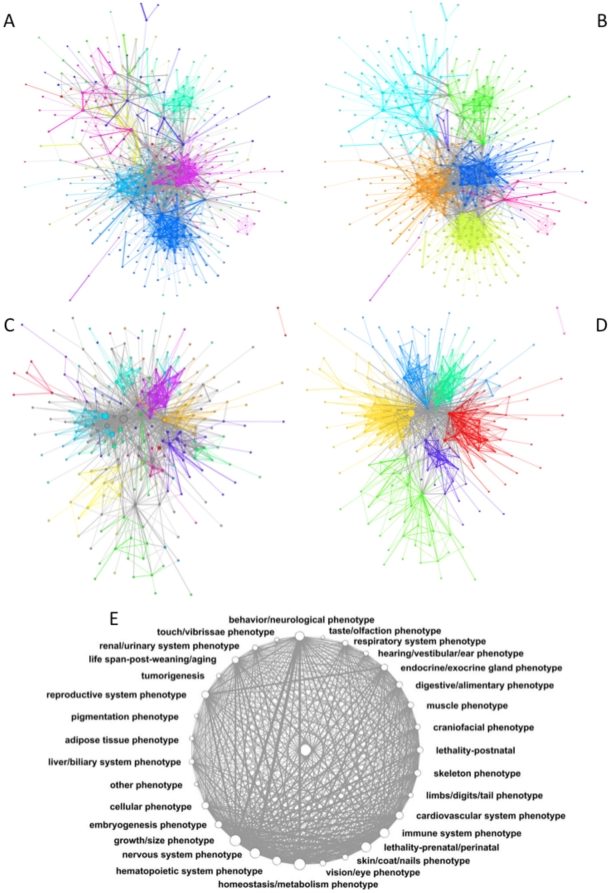
Visualisation of Gene and phenotype networks and communities. A) Gene network at MP level 5 using a threshold of *d*_cutoff_ = 0.009. Colouring corresponds to the highest-scoring MP term at level 1 in the weighted biadjacency matrix. Vertices with more than one equally high-scoring term are coloured in light grey. B) Gene network at MP level 5 using *d*_cutoff_ = 0.009 with colours indicating the communities extracted with Newman's spectral method. C) Phenotype network at MP level 5 using *d*_cutoff_ = 0.005. Colouring shows the most frequent parent MP term at level 1 if the MP DAG is traversed. Vertices with more than one of these were coloured white. D) Phenotype network at MP level 5 using *d*_cutoff_ = 0.005 with colours indicating the communities extracted with Newman's spectral method. E) Visualisation of the phenotype network at MP level 1 constructed with no *d* cut-off (*d*_cutoff_ = 0). This results in a highly-connected network. A&B: Vertices correspond to genes. Vertex size is proportional to the sum of scores in the weighted gene×gene adjacency matrix, which corresponds to the amount of association it has with other genes. Edges are proportional to the strength of association (edge weight in the adjacency matrix). B&C: Vertices correspond to MP terms in the MP. Vertex size is proportional to the sum of scores in the weighted phenotype×phenotype adjacency matrix.

Global views of the mouse gene-phenotype network (GPN) constructed using level 5 MP terms and their associated Ensembl gene identifiers are illustrated in **Figure 1**. The composition of these networks depends on whether genes or phenotypes are considered, the MP level considered, and the cut-off value of the association weight *d* used to accept edges into the network (see Methods for definition of *d*). Different d cut-offs were applied for different networks and different ontology levels (see Methods). Applying these cut-offs greatly reduces the coverage of the gene set in the network compared to using no cut-off (i.e. *d*_cutoff_ = 0). We observed reductions from 4996 to 430 (level 5; *d_cutoff_* = 0.009) and 2625 to 378 (level 8; *d_cutoff_* = 0.011) for gene networks and from 1269 to 239 (level 5; *d_cutoff_* = 0.005) and 512 to 199 (level 8; *d_cutoff_* = 0.002) for phenotype networks.

**Figure 1 (A&B)** shows the GPN as a gene network linked by phenotype terms. **Figure 1A** labels genes with their highest-scoring level 1 MP term to illustrate clustering of similar phenotypic outcomes within the network (see Materials and Methods for a description of the scoring process used to assign these terms), while **Figure 1B** is colour-coded according to communities (clusters) identified by the Newman spectral method [21]. **Figure 1 (C&D)** shows this network as a phenotype network linked by genes. Again, colour-coding is by Level 1 MP term in **Figure 1C** and Newman community in **Figure 1D**.

For both gene and phenotype networks there is a close but not perfect correspondence between Newman communities and regions of the graph coloured according to most common Level 1 MP term, suggesting that the GPN has a broadly modular nature. This is illustrated using a small number of sub-networks of interest in the Supplementary material (**Figure S2**). The full underlying data are presented as electronic supplementary text (**Text S1 & S2**). Corresponding networks constructed using Level 8 MP terms are shown in **Figure S3**.

**Figure 2:**
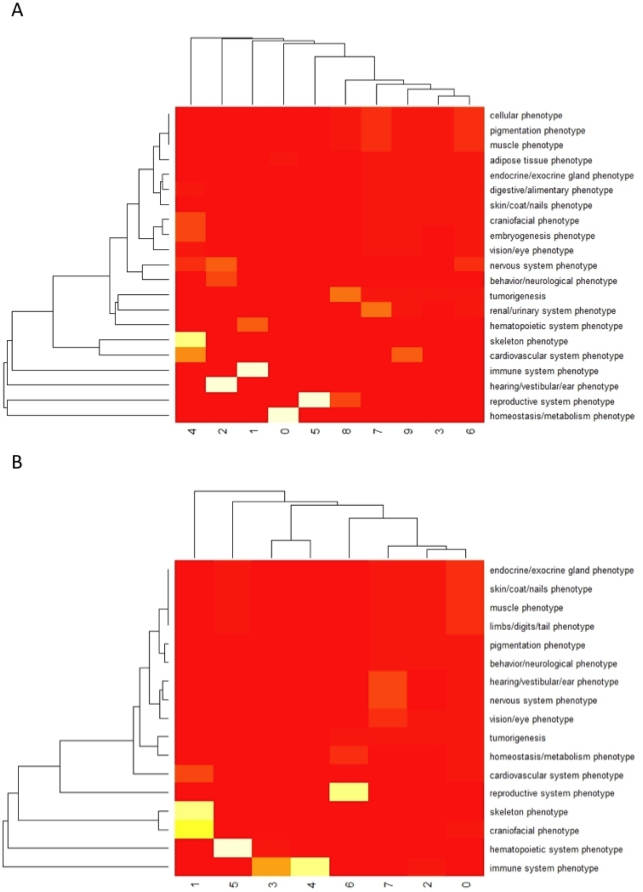
Mapping of communities to their most probable MP level 1 community based on gene membership overlap. Cells represent −log(p) where p is the probability of assignment based on overlap of the communities in the gene network with the community membership based on the most frequent MP level 1 membership. Red areas represent a low value and yellow areas represent high values. Dendrograms on the vertical axes represent hierarchical clustering using euclidean distances. A) MP level 5 communities; B) MP level 8 communities.

The correspondence between Newman communities and MP terms is shown clearly in the heat map representations shown in **Figure 2**, which represent Level 1 MP term frequencies across Newman communities. Some communities, notably communities 0, 5, 2 and 1, and to a lesser extent 4, showed a close mapping to individual MP terms. Others, however, showed only weak mappings to single MP terms and some little or no such mapping. Conversely, ten Level 1 MP terms showed no assignation to communities. MP Level 1 terms which form discrete communities are shown in **Table S1**.

### Topological properties of the GPN

**Figure 3:**
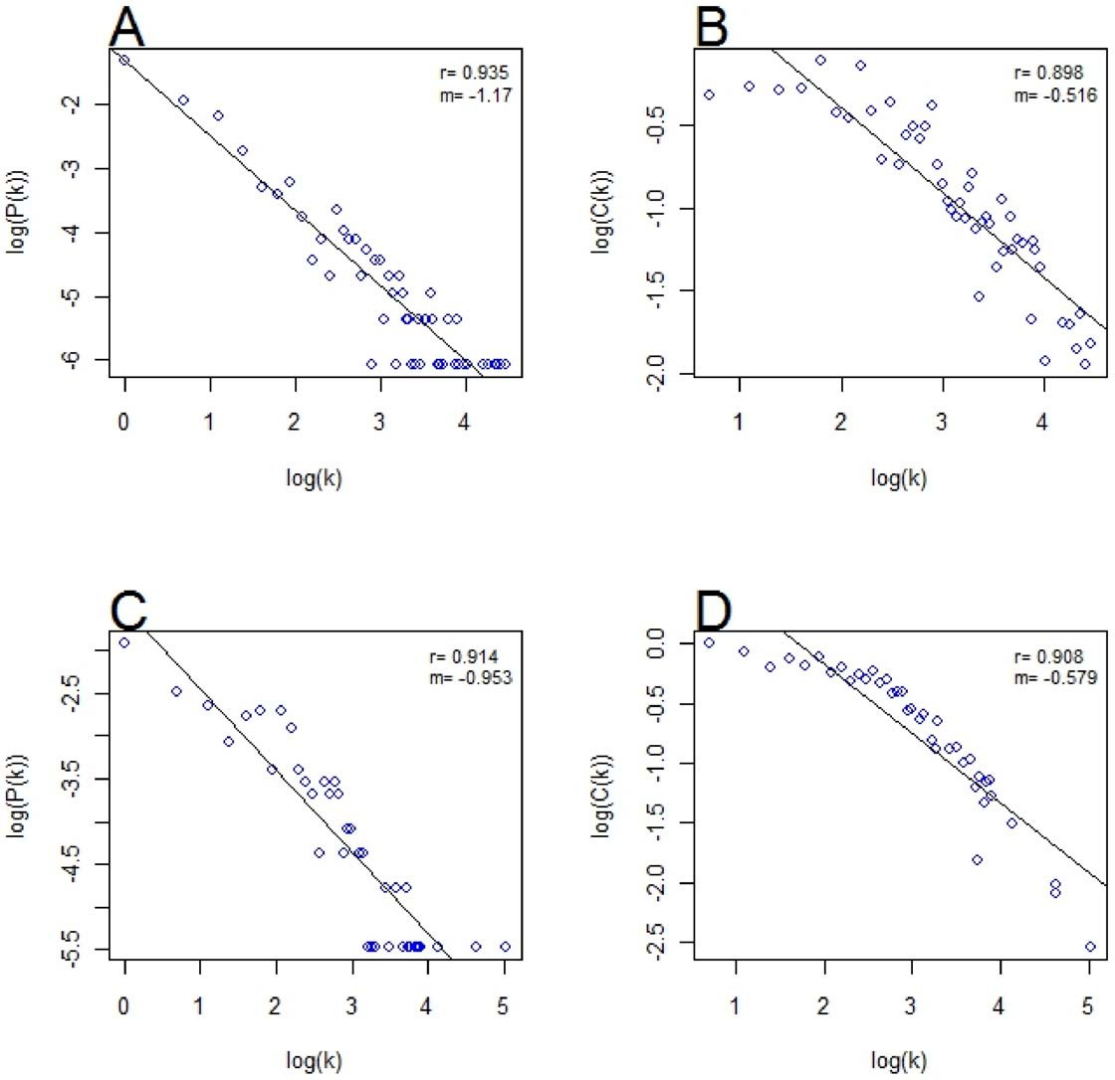
Topological properties of networks gene and phenotype networks at MP level 5. A) Gene network degree distribution and B) clustering coefficient distribution for the gene network at MP level 5, *d*_cutoff_ = 0.009. C) Phenotype Network degree distribution and D) clustering coefficient distribution for the phenotype networks at MP level 5, *d*_cutoff_ = 0.005. Black lines indicate a line of best fit using linear regression.

Biological networks characteristically have a scale-free and hierarchical nature [22]. The gene network at level 5, when parsed using a *d*_cutoff_ of 0.009, showed both of these properties (**Figure 3 A–B**). Scale-freeness is indicated by the linear nature of the log-log plot of P(k) against k (**Figure 3A**), while hierarchical nature is indicated by the linear log-log plot of C(k) vs k (**Figure 3B**) [22]. Corresponding figures to **Figure 3 (A & B)** for the network constructed using level 8 MP terms are presented in **Figure S4**.

When no cut-off is applied (*d*_cutoff_ = 0) the gene networks showed reduced scale-free character, switching to a mixed degree distribution type, i.e. containing varying proportions of scale-freeness and random network character (represented by normally distributed values of k) (**Figure S5, A–B, D–E, G–H**). The network constructed using level 1 MP terms was almost random in nature. The hierarchical character of the network is retained at level 1 with *d*_cutoff_ = 0 but gets progressively lost at lower levels (**Figure S5, C,F,I**).

The phenotype network at level 5 also showed a scale-free character and weak hierarchical character (**Fig. 3 C–D**). Again, the log-log plot of C(k) vs k was non-linear, as seen for the gene network. At *d*_cutoff_ = 0 the scale-free character was lost as for the gene network but a weak hierarchical character remained (**Figure S6**). A phenotype network constructed for Level 1 MP terms showed very high connectivity, most likely indicative of extensive pleiotropy in the system (**Figure 1E**).

### Modularity in the GPN

Visualisation of the level 5 gene network at *d*_cutoff_ = 0.009 results in the appearance of visually discernable modules (**Figure 1A**), consistent with a functional modularity in the mouse genetic machinery. Newman's algorithm [21] identified 10 communities in the level 5 gene network (**Figure 1B**) and 8 communities in the level 8 gene network. MP terms also naturally arrange themselves into visibly discernible clusters in the level 5 phenotype network (**Figure 1C**). In this case Newman's algorithm gave 7 communities in the level 5 network (**Figure 1D**) and 9 communities in the level 8 network (**Figure S3 and Table S1**).

To test if the modular character of the gene and phenotype networks was significantly greater than that of randomly wired networks we simulated randomly re-wired versions of the networks that kept the same node degree distribution (n = 1000). The empirical networks showed significantly higher clustering coefficients and Newman's Q values compared to these simulated networks (p≪0.001 in all four cases) (**Figure S7**). A stringent threshold on edges (on the order of d≥0.005–0.009) is needed to produce visually or computationally discernible communities in the mouse GPN (compare, for example, **Figures 1C and 1E**).

### Functional enrichment and phylogenetic profiles of communities and hubs extracted from the gene network

**Figure 4:**
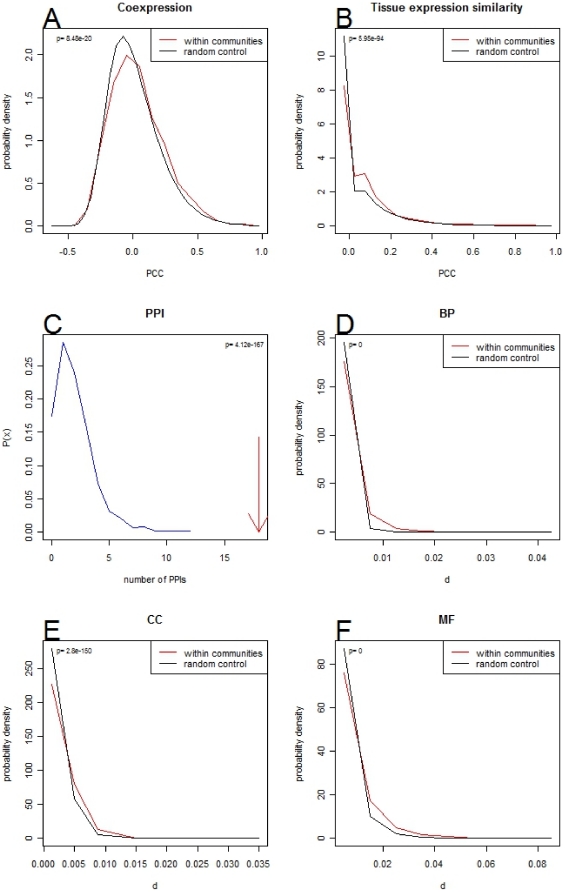
Functional enrichment in Gene networks. A) Coexpression increase measured by Pearson's correlation coefficient for genes within communities (red) compared to a random control (black) for the gene network at level 5 shown in Fig. 1 (A&B). B) GXD Tissue expression similarity increase for genes within communities (red) compared to a random control (black) for the gene network at level 5 shown in Fig. 1 (A&B). The similarity metric used was Pearson's correlation coefficient. C) Number of physical protein-protein interactions for genes within communities (red) compared to a random control (black) for the gene network at level 5 shown in Fig. 1. D–F) GO annotation similarity using the most common level for each of the GO ontologies for genes within communities (red) compared to a random control (black) for the gene network at level 5 shown in Fig. 1. The levels used were 5, 5, and 2 for BP, CC and MF respectively. The similarity metric used was the *d* score obtained using the same method as for the gene-MP term networks.

Goh et al [8] identified significant overrepresentation of coexpressed genes and gene interactions at the protein level in disease-associated modules in a human disease-gene network. To test if the communities in the gene network at *d*_cutoff_ = 0.009 correspond to components of similar functional modules we tested them for various measures of functional enrichment. Communities showed higher co-expression compared to random controls both for microarray RNA levels (**Figure 4A**) (p≪0.001) and tissue expression patterns in GXD (**Figure 4B**) (p≪0.001). They also show increased likelihood of having a physical interaction (**Figure 4C**) (p≪0.001) and increased GO term annotation similarity at the relevant level of each sub-ontology (**Figure 4D–F**) (p≪0.001 for all three branches of the GO).

We hypothesised that genes that are highly connected within their communities could act as regulators of function. To test this hypothesis, we took the hub genes (defined as genes having more connections than shown by 80% of all genes) from the gene network at level 5 and investigated the overrepresentation of GO terms within them. We found highly significant overrepresentation of GO:0016563 “transcription activator activity” (p<0.001) and weaker overrepresentation of GO:003682 “chromatin binding” (p<0.05), suggesting that highly connected nodes within communities tend to be involved in transcription regulation.

Functionally related proteins are believed to have correlated patterns of gain and loss during evolution [23], [24]. To test the hypothesis that the genes in the gene communities identified here coevolved we constructed phylogenetic profiles for all genes in mouse using the Ensembl Compara database [25]. Genes within communities showed a shift to higher correlation of phylogenetic profiles compared to non-community genes (p≪0.001) (**Figure S8**) and this effect was stronger for Level 5 than level 8 MP terms.

### Level 1 MP Term Enrichment within Gene Communities

If genes within communities tend to be functionally correlated, we would expect them also to share similar phenotypic outcomes, represented as MP terms. To test this we examined the numbers of genes within gene or phenotype networks that shared or did not share level 1 MP terms, taking into account whether or not they were members of the same Newman community. Results of these analyses are summarised in **Table 1**. Communities generally showed a modest overrepresentation of genes with the same level 1 MP term. In terms of gross numbers, this excess generally gave rise to highly significant Chi-squared values.

**Table 1:** Frequencies with which gene pairs within Newman communities share level 1 MP terms compared to frequencies for all genes.

Network	Same within communities	Same overall	P(Chi Sq, 3dof)
Level 5, Genes	0.64	0.54	≪0.001
Level 5, Phenotypes	0.41	0.29	≪0.001
Level 8, Genes	0.71	0.72	NS
Level 8, Phenotypes	0.22	0.19	<0.001

### Comparison of mouse communities to human communities

**Figure 5:**
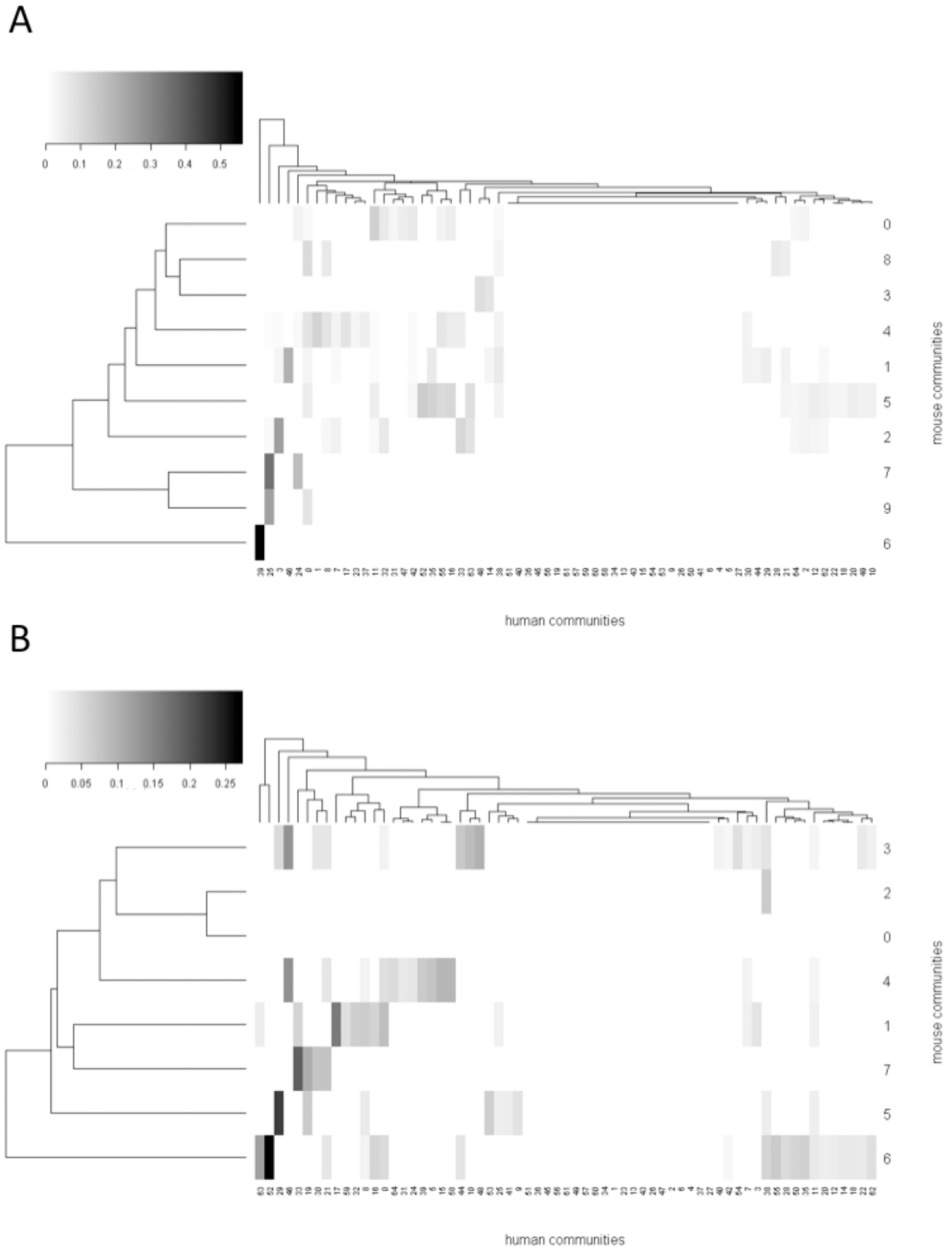
Mapping of MP and human disease communities to show the signifiance level of their gene membership overlap. Cells on the heatmap represent the significance of the overlap of the communities in the GPN and the disease-gene network. The intensities correspond to *−log(p)* where *p* is the probability of the observed overlap under the null hypothesis of random assignment of genes to communities. Dark areas have the highest values of *−log(p)* while white areas represent a low value. Numbers along the x-axis are the reference numbers of the Newman gene communities identified in the disease-gene network and numbers on the y-axis are the reference numbers of the Newman communities identified in the GPN. A) MP level 5 communities; B) MP level 8 communities.

To assess the extent to which our communites exhibited overlap with the communities from [8], we applied Newman's method to Goh et al's human disease gene network and calculated the extent of overlap between communities in the two networks. The results of the pairwise comparisons are shown in **Figure 5**. There are varying degrees of overlap between the two sets of communities. In general, the extent of overlap is relatively high if missing data is taken into account; the highest overlap scores reach approximately 0.6. However about half of the human communities had no apparent equivalents in the mouse communities, perhaps as a result of missing data. Comparing community mappings for the MP level 5 and level 8 networks (**Figures 5A & B**) shows more high-overlap matches for the MP level 8 network than for the level 5 network.

The level 5 community that corresponded most closely to a disease network community was community 6, which comprised four genes whose orthologues are associated with McKusick-Kaufman syndrome (MKKS) and the Bardet-Biedl syndrome (BBS) [26] and showed overrepresentation of the MP terms “cellular phenotype”, “pigmentation phenotype”, “muscle phenotype” and “nervous system phenotype”. This overlapped strongly with community 39 of the disease network, five of whose components were MKKS/BBS genes, including three MP community 6 genes (75%). Other MP communities showed broader mappings to a number of disease communities (**Figure 5**), **Table 2** presents the most common level 1 MP term associated with each phenotype community along with the disease term associated with the most strongly overlapping disease gene community for the MP Level 5 GPN. There is generally a good agreement between the two sets of terms. More detailed listings of gene community memberships for the MP Level 5 network are given in **Dataset S1**.

**Table 2:** Relationship between Level 1 phenotype terms associated with gene communities in the Level 5 network and the disease term associate with the most strongly overlapping disease gene network.

Level 5 Community No	MP term	Disease term of most similar disease community
0	Homeostasis/metabolism	Diabetes mellitus
1	Immune system	Severe combined immunodeficiency
2	Hearing/vestibular/ear	Deafness
3	Pigmentation/cellular/muscle	Atopy, Ataxia
4	Skeleton	Holoprosencephaly
5	Reproductive system	Fanconi anemia
6	Nervous system	Bardet-Biedl syndrome
7	Renal/urinary system	Hypertension
8	Tumorigenesis	Colon cancer
9	Cardiovascular system	Hypertension

### Hierarchical clustering of Level 1 MP terms

**Figure 6:**
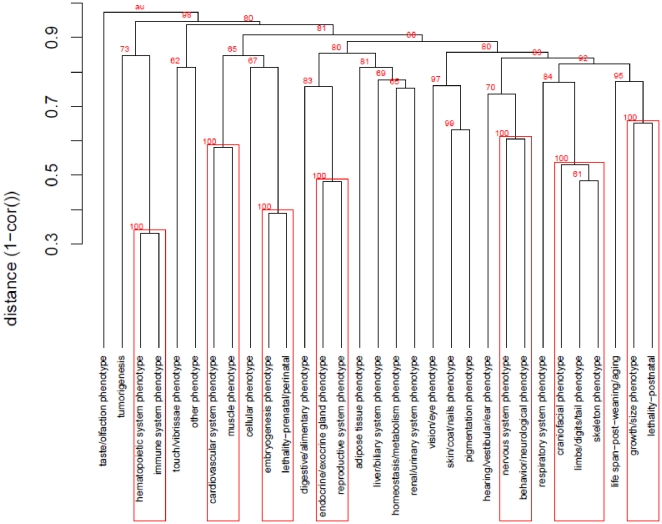
Hierarchical clustering of MP level 1 phenotypes according to gene commonality. Using the biadjacency matrix of genes×MP terms at level 1, a dendrogram was made using 1-cor() as a distance and average linkage as the agglomerative hierarchical clustering method (n*n*_bootstrap_ = 1×10^4^). Values in red indicate the approximately unbiased (AU) values associated with the cluster.

Given the relationship we observed between gene function and MP terms we investigated whether information contained in the network might be used to add structure to phenotype ontologies. We carried out clustering of Level 1 MP terms according to shared genes, assessing the significance of the clusters using approximately unbiased p-values (AU) [27] (**Figure 6**). This produced seven clusters with AU = 1.00 and four additional clusters of AU≥0.95 (including one which distinguished all phenotypes from MP:0005394 “taste/olfaction phenotype”). As illustrated in **Figure 6** the composition of these clusters was generally in line with biological intuition.

## Discussion

### Structural features of the mouse gene-phenotype network

In order to investigate the relationships between genes and phenotypes, and in particular the potential for systematic mouse phenotyping to identify underlying genetic pathways, we constructed a bipartite network linking Mammalian Phenotype ontology terms (MP terms) to associated genes using data from the Mouse Genome Database [2], which contains manually annotated data on mouse phenotypes taken predominantly from the literature, and the EuroPhenome database [7], which is a database of raw phenotyping data emerging from a number of systematic phenotyping projects, notably EUMODIC (http://www.eumodic.org) and assigned MP terms based on significant deviation of the results of individual tests from results on control animals [28]. Integration of these diverse data sets was only possible because of a shared use of the MP for annotation. We have confined ourselves to phenotype terms and gene identifiers in this analysis as we wished to study this relationship specifically. We did not consider individual alleles of genes (or their respective genetic backgrounds) as our aim was to link genes to phenotypes via their molecular products. Larger, more complex structures can be constructed to include gene function information, gene expression, protein-protein and gene-gene interactions and so on [17], [18], [19]. Such structures have been successfully used to predict some phenotypes resulting from gene perturbations in *Saccharomyces cerevisiae*, *Caenorhabditis elegans* and *Arabidopsis thaliana* [14], [15], [16].

Biological networks are typically scale- free and hierarchical [22]. That is, they contain a higher proportion of highly-connected nodes than a random network (scale-free) and are composed of separable modules, or communities (hierarchical). These properties are also seen in the mouse GPN but they are only seen if a relatively stringent cutoff is applied to remove weak connections (d≥0.009 for the gene network; d≥0.005 for the phenotype network). If weak connections are included a highly-connected graph lacking scale-freeness and with little hierarchical structure is produced. This is consistent with the widely-held view that many genes are pleiotropic, i.e. have effects on many different phenotypes. Only by characterising the strongest gene-phenotype links can we identify a more typical network organisation.

The parameter 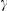, which is the exponent of the relationship between the probability of observing k connections at a node and the connection number 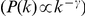, is an important property of biological networks [22]. Networks with values of 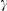 below 3 have been shown to be dominated by hubs and have a high degree of robustness to loss of individual nodes at random [22]. Both the phenotype and gene networks derived from the mouse GPN had 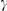 of the order of 1 (**see** **Figure 3**), suggesting that both networks are robust and dominated by highly connected hubs. A further property of biological networks is that they are hierarchical, consisting of highly connected modules linked by less connected nodes [22]. Hierarchical networks are characterised by a linear relationship of the log-log plot of C(k), the clustering coefficient of a node, and k, the node degree as seen in **Figures 3**, **S4 and S5** for both the gene and phenotype networks we describe here. We conclude that both the gene and phenotype networks are hierarchical in nature when constructed using sufficiently stringent d cut-offs. It should be noted, however, that we observed that both the scale-freeness and hierarchical nature of the networks are lost as lower stringency connections are used, resulting in the limit in a highly interconnected structure (**Figure 1E**). This may again reflect the highly interconnected (pleiotropic) nature of the genetic networks underlying phenotypes, with many genes contributing, albeit weakly, to many phenotypic outcomes.

In characterising their human disease-gene network, Goh et al [8] showed that genes associated with similar disorders show an increased propensity to mutual physical interactions and gene coexpression. We investigated whether the gene communities within the GPN showed similar features by considering gene coexpression, tissue coexpression patterns in GXD, protein-protein interaction and GO terms. In all cases we found highly significant overrepresentation within modules, as would be expected if the genes associated with particular MP terms were themselves associated with specific underlying processes that give rise to abnormal phenotypes when disrupted. We could also show higher correlations of phylogenetic profiles within communities than between non-community genes. This suggests that groups of genes giving rise to particular characteristics of organisms have coevolved, consistent with the observation that groups of interacting proteins tend to show coevolutionary patterns of gene duplication and sequence divergence [24].

As scale-free networks are dominated by hubs, and in particular in light of evidence of gene coexpression within gene communities, we characterised the network hubs (defined here as the top 20% most connected nodes) in terms of their Gene Ontology annotations. We found a strong overrepresentation of the term “transcription factor activity” in hubs, indicating that the behaviour of individual communities may be regulated by particular transcription factors and that these transcription factors may be responsible for regulating the expression of particular phenotypes. Yu et al [29] have argued that transcription factors with many target genes tend to be essential genes, while essential genes tend to be hubs in gene networks [8], [30]. Thus high level phenotypes may be controlled by a subset of transcription factors which may have multiple targets and be essential. Interestingly, around one third of gene knockouts in the Knockout Mouse Project ES cell library characterised so far are embryonic lethal [1]. It will be interesting to characterise these genes further as the data set grows.

### Gene communities and phenotype diffuseness

Whereas Goh et al [8] selected gene sets based on their disease annotations we used an objective criterion [21] for identifying modules within the GPN in this study. A benefit of this approach is that it avoids ascertainment bias due to lack of data on certain phenotypic domains, which is a well-known problem of datasets not derived from systematic phenotyping, such as MGD. A disadvantage of this approach is that only 10 communities are observed within the level 5 gene network that roughly correspond to particular level 1 MP terms, although there are 31 level 1 phenotypes in MP. In particular, whereas “immune system phenotype”, “hearing phenotype” and “reproductive system phenotype” were always resolved into very tight groups this was not the case for other terms. Further analysis of the networks (see **Figure 2**) suggests that other level 1 phenotypes are more “diffuse” in their distribution across the network.

In comparing our networks with Goh et al's [8] disease-gene network we found a number of good correspondences between the gene sets making up Newman communities in the two networks, especially at the level of MP term and disease annotations (see Figure 5; Table 2). The Newman communities in the disease-gene network do not exactly correspond to diseases but we could identify the most common disease term for each community and relate it to the most common MP term in the MP term-gene networks. This comparison reveals a good match between MP terms and disease terms (Table 2). The mappings in Table 2 to some extent resemble the phenologs described by McGary et al [31]. Phenologs are phenotypes that are considered homologous between species and defined by groups of orthologues which are shared between phenotypes in different species. Our mappings differ from phenologs as they are defined using gene communities identified objectively in the network rather than sets of genes annotated to a given phenotype.

The comparison in Figure 5 indicates that although individual communities may map quite well, MP terms do not map directly onto individual diseases. This is unsurprising as human diseases are typically characterised by a constellation of atomic phenotypes [4]. Mapping between disease terms (or disease endophenotypes) and MP terms may be possible using networks of this kind however. In this analysis we identified only ten communities in the GPN at MP level 5 and eight at MP level 8, whereas many more could be identified in the disease-gene network. Adding additional data to the GPN may give rise to more communities, allowing a closer mapping between mouse and human phenotype sets. Interestingly, we observed more strong overlaps between phenotype and disease communities when we considered level 8 MP terms than with level 5 MP terms. This deserves further analysis but may suggest that it will be important to collect granular phenotype data in systematic phenotyping programmes to allow linking of individual mouse models to diseases.

The diffuse distribution of many level 1 MP terms in the gene network (Figure 2) could be due to a number of not necessarily mutually exclusive factors. It could in part represent the incompleteness of the data set, both in terms of the genes that have been characterised (N<5,000 annotated at MP level 5 or higher) and the amount of phenotype information available for individual genes – the bulk of the information represented in the mouse GPN we present here comes from individual, *ad hoc* studies which focus not only on individual genes but also on individual phenotype domains, meaning that much phenotypic information is likely to be missing from the data set. To eliminate this incompleteness there is a strong argument for collecting systematic phenotype data on as many mouse genes as possible [1] at as high a granularity as possible.

Two other causes might contribute to the relative diffuseness of many level 1 MP terms in the mouse GPN. Firstly, it might reflect the more highly pleiotropic nature of these phenotype classes. If this were the case it would suggest that forward genetic approaches which make use of these more diffuse phenotypes will have more difficulty in identifying consistent sets of underlying genes (e.g. pathways). For some of the level 1 MP terms that do not associate strongly with gene communities this seems reasonable – for example “growth/size phenotype”, “lethality-postnatal”, “lethality-postnatal” or “life-span-post-weaning/aging”. Other categories, however, might be expected to have a clear genetic basis, for example “taste/olfaction phenotype” or “touch/vibrissae phenotype”. These latter classes however have the fewest genotype annotations in the MGI database (143 and 546 genotypes respectively on 27.07.10) so it is possible that they had too few connections to form significant communities in the GPN.

A third source of the diffuseness of many level 1 MP terms across the GPN could be the structure of the MP ontology itself, as it could reflect a lack of alignment between the phenotypes represented at high levels of the ontology and underlying genetic processes. The high level terms in the MP reflect mouse biologists' intuitive views of the categories into which phenotypes should be grouped. This does not necessarily bear any relationship to the underlying processes giving rise to these phenotypes. Level 1 MP terms which are diffuse on the mouse GPN might then be unnatural groupings in the sense that they do not reflect underlying processes. Perhaps, for example, the lower level terms that make them up should be split into more than one higher level term.

What might an ontology that more closely aligned phenotypes to genetics look like? As a first attempt to address this we clustered level 1 MP terms by the proportion of the underlying genes they share. The result of this is illustrated in **Figure 6**. This structure contains a number of groupings of Level 1 terms not present in the ontology itself. These high-level clusters are generally consistent with biological intuition, suggesting that such an approach might be of value in giving a more realistic structure to high level MP terms.

### Conclusions

We have shown that constructing a mouse gene-phenotype from published data allows us to identify functional modules related to high-level phenotype terms in the Mammalian Phenotype ontology. This supports the hypothesis that the mammalian genome is organised into interconnected modules of physiological and cellular function that have a direct read-out at the phenotype level. This in turn indicates that systematic phenotyping in mouse and other model organisms [1], [32] has the potential to identify protein complexes, coexpressed sets of genes or molecular pathways underlying many phenotypes.

Our findings are mirrored in studies on other model organisms such as that by Gunsalus et al in *C. elegans* [33] in which phenotypic similarity of RNAi knockdowns of cellular defects in early embryogenesis correlated positively with GO term sharing. In the same study, phenoclusters of genes with correlated phenotype profiles were constructed and intra-phenocluster pairs were found to be functionally enriched in specific GO functions, displayed enriched amounts of physical protein-protein interactions, and showed enriched expression correlation. A combined network of phenotypic similarity, expression similarity and protein-protein interaction parsed for retention of only edges with two or more types of function gave topological clusters that predicted shared GO terms with high specificity. Eight out of ten proteins with unknown function that were experimentally checked for localisation were consistent with the predicted function. Studies in human cells such by Fuchs et al [34], in which genes were clustered by high-throughput cellular morphology screens, also provided phenotypic clusters that were predictive of gene function and could be used to identify specific functions for several genes.

In yeast, the model organism for which the largest number of genetic interactions have been studied, comprehensive interaction networks were made using a synthetic genetic array that automated the isolation of double mutants [35]. The density of the resulting interaction map mirrors our results, in which the gene networks of phenotypic similarity are very densely connected for high levels of the MP, suggesting greater connectivity which transcends physical interactions [35]. Genes connected inside this yeast gene network often shared GO term annotations and the network could be used to predict protein-protein interactions.

In this study, a number of high-level terms in the MP show a diffuse distribution across the network, suggesting that systematic phenotyping may find it more difficult to identify underlying pathways for these classes of phenotype than for less diffuse phenotype classes. This is a particular issue for high throughput phenotyping programmes such as EUMODIC [1] which seek to relate phenotype to gene function. The diffuseness of phenotypes could have a number of causes, including limited or biased data sets, the general pleiotropy of genes underlying some of these phenotypes or malformed ontologies. The first two of these issues can be addressed by systematic phenotyping, which can add missing genes, especially for under-studied phenotypes, and add additional phenotypes to individual genes, increasing our understanding of pleiotropy. To address the third aspect it is possible that a reconfigured phenotype ontology, based on the GPN and specifically designed for phenotyping experiments, will be needed. We illustrate a possible approach to producing a phenotype ontology structure that more closely reflects the underlying genetic architecture.

Comparing communities between MP networks and the human disease gene network shows that there is a many-to-many relationship between the two types of community. However there appears to be a closer relationship between phenotype and disease communities if more granular levels of the MP are used, suggesting that high throughput phenotyping should aim for the most granular possible phenotype annotations to maximise their utility.

## Materials and Methods

### Gene-phenotype association datasets

Gene-phenotype associations and the MP ontology OBO file were downloaded from MGI [2] via the ftp site on 10.2.2010. The downloaded files were: ftp://ftp.informatics.jax.org/pub/reports/MPheno_OBO.ontology and ftp://ftp.informatics.jax.org/pub/reports/MGI_PhenoGenoMP.rpt.

The MGI_PhenoGenoMP.rpt file, containing the lowest-level available annotations, was parsed to produce a non-redundant bipartite graph of gene-phenotype associations. Gene-phenotype associations obtained from Europhenome were sourced from a custom-made report of Europhenome gene IDs to MP term links extracted on 19.1.2010. This was also parsed with the files provided to produce a non-redundant bipartite graph of gene-phenotype associations. The two lists were amalgamated to form a single bipartite edge list of genes to Ensembl IDs. The MP terms “no phenotypic analysis”, “normal phenotype”, “no abnormal phenotype detected”, “phenotypic reversion”, “reversion by mitotic recombination”, “reversion by viral sequence excision” were excluded from the analysis since they were not considered to convey useful information. Gene dosage was disregarded such that if either a heterozygote or a homozygote showed a phenotype, the gene was considered to be associated with the MP term. The MGI marker IDs and other IDs were converted to Ensembl IDs using the Ensembl Perl API. Absence of annotation to an MP term could have two possible causes: either no data has been recorded to support a given annotation or, because MP does not allow for annotation of normal phenotypes, they may have been absent but not annotated. These two states were not distinguished during the construction of the gene-phenotype network as the relevant information is not available.

The GO OBO file was downloaded from the GO website on 10.2.2010. The downloaded file was: http://www.geneontology.org/ontology/obo_format_1_2/gene_ontology.1_2.obo. Gene-GO associations were downloaded from Ensembl using the Ensembl Perl API. OBO files were parsed with a Perl script to give computational representations of the DAG and the subsequent analysis of term inheritance.

### Construction of gene networks and MP term networks

We mapped all MP terms in the MGI MGI_PhenoGenoMP.rpt report to their levels in the MP hierarchy. To accommodate mixed ontology level annotations we elected to focus our analysis on the most commonly used level of annotation, which was level 5, and an arbitrarily chosen lower level, level 8, for more granular annotation.

### Gene-phenotype data matrices

A weighted measure of phenotype propensity was used so as to produce a weighted biadjacency matrix of genes and phenotypes. This matrix is dependent on the data and the phenotype ontology structure. Phenotypes were allocated a granularity weight such that a phenotype was considered more granular if there are more annotated nodes below it in the ontology. This was
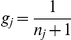
where *g_j_* denotes granularity of phenotype j and n_j_ denotes the number of terms below term j in the ontology that are annotated data set.

The weighted gene×phenotype biadjacency matrix B was calculated by considering the association of a gene with a phenotype as
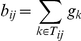
Where *T_ij_* are all the terms annotated for gene *i* that are children nodes of term *j* at any level below term j. This was done by using the raw gene-phenotype edge list and converting the MP term annotation to the desired level and adding the *g_j_* weights for each gene. Cases where MP terms contained multiple parents therefore produced multiple annotation contributions at higher levels.

### Association metric for genes and phenotypes

The pairwise association of two genes i_1_ and i_2_ for a particular phenotype j was defined as
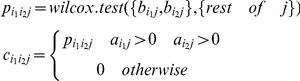
Where Wilcox.test() is a two-sample Wilcoxon test. The values from the tests were corrected using the fdrtool package in R [36]. A final score *d_i1i2_*, for two genes was defined as:
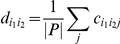
This is the average value of d over all phenotypes at that level. It gives a score in the interval [0,1] and served as a metric for gene associations for all gene pairs. The score was calculated for all gene pairs and thus produced a *i*×*i* matrix that represented the weighted adjacency matrix for the gene network.

Transposing the matrix A to give A^T^ gave the biadjacency matrix with genes as features of phenotypes. Repeating the procedure gave the weighted adjacency matrix for the phenotype network.

### Parsing of networks

To obtain only the strongest associations, the edge list of the gene and phenotype networks obtained from the two methods described above were parsed using at a particular d-value obtained by inspection of the distribution of d-values in the network's edges.

### Community extraction from networks

Communities were extracted from networks using Newman's spectral method [21]. A C program was adapted from one provided by the author and this adapted program was incorporated into the analysis pipeline.

### Hubs and hub GO enrichment

Hubs were defined as nodes with a degree in the top 20% of the node degree distribution in the network that they came from. We used a one-tailed Fisher's exact test comparing the GO terms in each community compared with the appropriate background. The background was taken to be all the genes in MGI for the appropriate level, in this case, level 5. The p-values were corrected using the fdrtool function from the fdrtool package in R [36] for each category.

### Gene expression enrichment

Expression data was obtained from the genome-wide microarray analysis of Zhang et al. [37] and from GXD [38], [39]. The microarray files were parsed and the correlation matrix was calculated. The GXD expression data was taken from a custom made report in the form of a binary (0,1) matrix of genes×tissues IDs and a list of tissue ID names.

To represent the associations within communities, a vector was made that consisted of the union of all pairwise gene associations within communities for each community. The background control was based on random re-sampling of gene communities of the same size sampled randomly from the appropriate original network's gene set (*n*_bootstrap_ = 1000). A two-sampled Wilcoxon test was used on the two vectors by applying the Wilcox.test() function in R.

### Protein-protein interactions

Physical protein-protein interactions (PPI) were taken from IntAct [40], Biogrid [41], MIPS [42] and DIP [43]. The files downloaded were: ftp://ftp.ebi.ac.uk/pub/databases/intact/current/psimitab/intact.txt, http://www.thebiogrid.org/downloads/datasets/NCBI.tab.txt and http://mips.gsf.de/proj/ppi/data/mppi.gz. All files were downloaded on 10.2.2010. The DIP data was downloaded manually on 22.4.2009.

The Biogrid file was parsed for physical interactions. The physical interaction experimental systems retained were: “Affinity Capture-MS”, “Affinity Capture-RNA”, “Affinity Capture-Western”, “Co-fractionation”, “Co-localization”, “Co-purification”, “FRET”, “Two-Hybrid”, “Biochemical Activity”, “Co-crystal Structure”, “Far Western”, “Protein-peptide”, “Protein-RNA”, “Reconstituted Complex”.

The protein IDs were converted to Ensembl gene IDs using files obtained from Ensembl using the Perl API. The separate PPI edge lists were amalgamated to form a single PPI network.

A random control of the number of PPIs in communities was obtained by simulating random communities as previously described for the gene expression enrichment analysis. The numbers of interactions in random communities were amalgamated to produce a control compared to the real amount with a one-sampled Wilcoxon test.

### GO annotation similarity enrichment

Pairwise GO similarity scores for genes were obtained by using the adjacency matrix of the level containing the most annotations and which would provide a compromise between granularity and amount of annotations. The chosen levels were 5 for BP, CC and level 2 for MF. The score d was calculated from the biadjacency matrix for all gene pairs in an adjacency matrix as described for for the gene expression enrichment analysis.

Treatment and control vectors were obtained as described previously and a two-sampled Wilcoxon test was used as before to obtain significance.

### Phylogeny data

Phylogenetic data was taken from the Ensembl Compara database using the Perl API. The data from Ensembl gene tree objects were parsed to put them in the form of a gene×species matrix where the elements are integers denoting the copy number of the genes present in the species. Substituting the row names of this matrix (Ensembl tree IDs) for mouse genes gave the required phylogenetic profile matrix for mouse. The correlation matrix was calculated to obtain all pairwise gene correlations.

Treatment and control vectors for communities were obtained from this matrix as previously described.

### Assignment of gene communities to highest scoring MP level 1 groups

The assignment of gene communities to groups defined by highest scoring MP level 1 phenotypes was done by calculating the p-value for the probability of each community containing the overlap with each group defined by highest-scoring MP level 1 phenotype. All pairwise comparisons were made. The probability mass function that describes the probability of getting *z* elements in the intersection set Z is
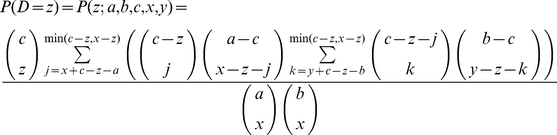
where
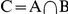


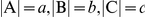
And we sample X from A of size *x* and a sample Y from B of size *y* such that
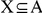


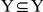


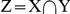


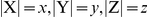
The cumulative distribution function (CDF) is
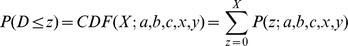
The probability of observing a value as least as extreme as the observed value is therefore given by
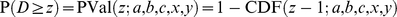
which gives the p-value as required. For graphical purposes the quantity −log(*p*) was used as this allows easier differentiation of p-values. Because community sizes were small in this case, this probabilistic metric could be calculated but it was computationally intractable for larger data sets.

### Comparison of mouse communities to human communities

Data from Goh et al were downloaded from http://www.pnas.org/content/suppl/2007/05/03/0701361104.DC1/01361Table2.pdf

Newman's spectral method was applied to extract the communities. An all-against-all comparison of the communities was computed using a metric of overlap, *p*, such that
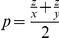
where *z* is the amount of genes overlapping in the two communities, *x* is the amount of genes in the first community and *y* is the amount of genes in the second community. This is the average of the fraction of common elements for each set.

### Clustering of high-level phenotypes

The gene×phenotype biadjacency binary matrix A described above for MP level 1 annotations was used to cluster the phenotypes using the Parpvclust package in R [44], which uses R's hclust function to carry out agglomerative hierarchical clustering. This package calculates approximately unbiased (AU) bootstrapping to assess the uncertainty in the clustering. The distance method used was 1-cor(), where cor() is the correlation operator in R, which calculated Pearson correlation coefficients. The average linkage method was used and n_boot_ = 1×10^4^.

## Supporting Information

Figure S1 Probability mass for the frequency of annotations at different levels of the MP in the amalgamated list of gene-phenotype associations from MGI and Europhenome and the three GOs in Ensembl.
(TIFF)

Figure S2 Details of example gene communities: A) Community 2 in the MP level 5 gene network. This community visually corresponds to the community that is frequently annotated with the granularity level 1 MP term “hearing/vestibular/ear phenotype” [MP:0005377] and is highly enriched in the GO terms “mechanoreceptor differentiation” [GO:0042490] and “sensory perception” [GO:0007600]. B) Community 5 in the MP level 8 gene network. This community visually corresponds to the community that is frequently annotated with the granularity level 1 MP term “hematopoietic system phenotype” [MP:0005397] and is highly enriched in the GO terms “erythrocyte homeostasis” [GO:0034101]. Details of example phenotype communities: C) Example of a phenotype community at level 5, illustrating a community of phenotypes related to diabetes (in blue). D) Example of a phenotype community at level 8 illustrating a community pf phenotypes related to reproductive system abnormalities (in green).
(TIFF)

Figure S3 Gene and phenotype networks at MP level 8. Attributes are as in Fig. 1 A–D.
(TIFF)

Figure S4 Topological analysis plots of level 8 networks, with cut-off. A)–B) Gene network degree distribution and clustering coefficient for the gene network at MP level 8, *d*_cutoff_ = 0.011. C)–D) Phenotype Network degree distribution and the clustering coefficient distribution for the phenotype network at level 8, *d*_cutoff_ = 0.002. Black lines indicate a line of best fit using linear regression.
(TIFF)

Figure S5 Topological analysis plots of level 5 gene networks, with no *d* cut-off. A–B) Gene network degree distribution and C) clustering coefficient distribution for the gene network at MP level 1, *d*_cutoff_ = 0. D–F) The same quantities for MP level 5. G–I) The same quantities for MP level 8. Black lines indicate a line of best fit using linear regression.
(TIFF)

Figure S6 Topological analysis plots of phenotype networks with no cut-off. A–B) Gene network degree distribution and C) clustering coefficient distribution for the gene network at MP level 5, *d*_cutoff_ = 0. D–F) The same quantities for MP level 8.
(TIFF)

Figure S7 Modularity in the gene and phenotype networks. A)–B) Average clustering coefficient and Newman's modularity (Q) for randomly re-wired networks (blue) and the corresponding gene network at MP level 5 shown in Fig. 4.*d*_cutoff_ = 0.011. The random networks retain the same degree distribution as the empirical gene network. C)–D) Average clustering coefficient and Newman's modularity (Q) for randomly re-wired networks (blue) and the corresponding phenotype network at MP level 5 shown in Fig. *d*_cutoff_ = 0.005.
(TIFF)

Figure S8 Phylogenetic profile correlation in communities. Shown are probability densities for genes within communities (red) compared to a random control (black) for the gene networks presented in the text at level 5 (A) and 8 (B).
(TIFF)

Text S1 Edge list for the gene network at MP level 5 and with d_cutoff_ = 0.009.
(TXT)

Text S2 Edge list for the phenotype network at MP level 5 and with d_cutoff_ = 0.005.
(TXT)

Table S1 Top level MP terms that can form discrete communities, form communities which fall below the stringency cut-off (P<0.0001), or do not form communities. Communities are assessed within the phenotype networks constructed at MP levels 5 and 8, respectively.
(PDF)

Dataset S1 The first worksheet shows the compositions of Newman communities detected in the GPN at MP level 5 and in the disease-gene network (showing both human gene composition and mouse orthologue composition). The second worksheet shows the genes in the GPN communities that overlap with members of disease-gene communities, and the disease-gene communities they are members of.
(XLSX)

## References

[1] Brown SD, Wurst W, Kuhn R, Hancock J (2009). The Functional Annotation of Mammalian Genomes: The Challenge of Phenotyping.. Annu Rev Genet.

[2] Bult CJ, Kadin JA, Richardson JE, Blake JA, Eppig JT (2010). The Mouse Genome Database: enhancements and updates.. Nucleic Acids Res.

[3] Hancock JM, Mallon A-M (2007). Phenobabelomics–mouse phenotype data resources.. Brief Funct Genomic Proteomic.

[4] Schofield PN, Gkoutos GV, Gruenberger M, Sundberg JP, Hancock JM (2010). Phenotype ontologies for mouse and man; bridging the semantic gap.. Disease Models & Mechanisms.

[5] Hancock JM, Mallon AM, Beck T, Gkoutos GV, Mungall C (2009). Mouse, man, and meaning: bridging the semantics of mouse phenotype and human disease.. Mamm Genome.

[6] Smith CL, Goldsmith CA, Eppig JT (2005). The Mammalian Phenotype Ontology as a tool for annotating, analyzing and comparing phenotypic information.. Genome Biol.

[7] Morgan H, Beck T, Blake A, Gates H, Adams N (2010). EuroPhenome: a repository for high-throughput mouse phenotyping data.. Nucleic Acids Res.

[8] Goh KI, Cusick ME, Valle D, Childs B, Vidal M (2007). The human disease network.. Proc Natl Acad Sci U S A.

[9] Ashburner M, Ball CA, Blake JA, Botstein D, Butler H (2000). Gene ontology: tool for the unification of biology. The Gene Ontology Consortium.. Nat Genet.

[10] Butte AJ, Kohane IS (2006). Creation and implications of a phenome-genome network.. Nat Biotechnol.

[11] Jiang JQ, Dress AW, Chen M (2010). Towards prediction and prioritization of disease genes by the modularity of human phenome-genome assembled network.. J Integr Bioinform.

[12] Linghu B, Snitkin ES, Hu Z, Xia Y, Delisi C (2009). Genome-wide prioritization of disease genes and identification of disease-disease associations from an integrated human functional linkage network.. Genome Biol.

[13] Wu X, Jiang R, Zhang MQ, Li S (2008). Network-based global inference of human disease genes.. Mol Syst Biol.

[14] Lee I, Ambaru B, Thakkar P, Marcotte EM, Rhee SY (2010). Rational association of genes with traits using a genome-scale gene network for Arabidopsis thaliana.. Nat Biotechnol.

[15] Lee I, Lehner B, Crombie C, Wong W, Fraser AG (2008). A single gene network accurately predicts phenotypic effects of gene perturbation in Caenorhabditis elegans.. Nat Genet.

[16] McGary KL, Lee I, Marcotte EM (2007). Broad network-based predictability of Saccharomyces cerevisiae gene loss-of-function phenotypes.. Genome Biol.

[17] Kim WK, Krumpelman C, Marcotte EM (2008). Inferring mouse gene functions from genomic-scale data using a combined functional network/classification strategy.. Genome Biol.

[18] Pena-Castillo L, Tasan M, Myers CL, Lee H, Joshi T (2008). A critical assessment of Mus musculus gene function prediction using integrated genomic evidence.. Genome Biol.

[19] Guan Y, Myers CL, Hess DC, Barutcuoglu Z, Caudy AA (2008). Predicting gene function in a hierarchical context with an ensemble of classifiers.. Genome Biol.

[20] Groth P, Weiss B, Pohlenz HD, Leser U (2008). Mining phenotypes for gene function prediction.. BMC Bioinformatics.

[21] Newman ME (2006). Modularity and community structure in networks.. Proc Natl Acad Sci U S A.

[22] Barabasi AL, Oltvai ZN (2004). Network biology: understanding the cell's functional organization.. Nat Rev Genet.

[23] Pellegrini M, Marcotte EM, Thompson MJ, Eisenberg D, Yeates TO (1999). Assigning protein functions by comparative genome analysis: protein phylogenetic profiles.. Proc Natl Acad Sci U S A.

[24] Vinogradov AE (2010). Systemic factors dominate mammal protein evolution.. Proc R Soc B.

[25] Vilella AJ, Severin J, Ureta-Vidal A, Heng L, Durbin R (2009). EnsemblCompara GeneTrees: Complete, duplication-aware phylogenetic trees in vertebrates.. Genome Res.

[26] Schaefer E, Durand M, Stoetzel C, Doray B, Viville B (2010). Molecular diagnosis reveals genetic heterogeneity for the overlapping MKKS and BBS phenotypes.. Eur J Med Genet.

[27] Shimodaira H (2008). Testing Regions with Nonsmooth Boundaries via Multiscale Bootstrap.. Journal of Statistical Planning and Inference.

[28] Beck T, Morgan H, Blake A, Wells S, Hancock JM (2009). Practical application of ontologies to annotate and analyse large scale raw mouse phenotype data.. BMC Bioinformatics.

[29] Yu H, Greenbaum D, Xin Lu H, Zhu X, Gerstein M (2004). Genomic analysis of essentiality within protein networks.. Trends Genet.

[30] Jeong H, Mason SP, Barabasi AL, Oltvai ZN (2001). Lethality and centrality in protein networks.. Nature.

[31] McGary KL, Park TJ, Woods JO, Cha HJ, Wallingford JB (2010). Systematic discovery of nonobvious human disease models through orthologous phenotypes.. Proc Natl Acad Sci U S A.

[32] Houle D, Govindaraju DR, Omholt S (2010). Phenomics: the next challenge.. Nat Rev Genet.

[33] Gunsalus KC, Ge H, Schetter AJ, Goldberg DS, Han JD (2005). Predictive models of molecular machines involved in Caenorhabditis elegans early embryogenesis.. Nature.

[34] Fuchs F, Pau G, Kranz D, Sklyar O, Budjan C (2010). Clustering phenotype populations by genome-wide RNAi and multiparametric imaging.. Mol Syst Biol.

[35] Tong AH, Lesage G, Bader GD, Ding H, Xu H (2004). Global mapping of the yeast genetic interaction network.. Science.

[36] Strimmer K (2008). fdrtool: a versatile R package for estimating local and tail area-based false discovery rates.. Bioinformatics.

[37] Zhang W, Morris QD, Chang R, Shai O, Bakowski MA (2004). The functional landscape of mouse gene expression.. J Biol.

[38] Finger JH, Smith CM, Hayamizu TF, McCright IJ, Eppig JT (2010). The mouse Gene Expression Database (GXD): 2011 update.. Nucleic Acids Res.

[39] Ringwald M, Eppig JT, Kadin JA, Richardson JE (2000). GXD: a Gene Expression Database for the laboratory mouse: current status and recent enhancements. The Gene Expression Database group.. Nucleic Acids Res.

[40] Kerrien S, Alam-Faruque Y, Aranda B, Bancarz I, Bridge A (2007). IntAct–open source resource for molecular interaction data.. Nucleic Acids Res.

[41] Stark C, Breitkreutz BJ, Reguly T, Boucher L, Breitkreutz A (2006). BioGRID: a general repository for interaction datasets.. Nucleic Acids Res.

[42] Pagel P, Kovac S, Oesterheld M, Brauner B, Dunger-Kaltenbach I (2005). The MIPS mammalian protein-protein interaction database.. Bioinformatics.

[43] Salwinski L, Miller CS, Smith AJ, Pettit FK, Bowie JU (2004). The Database of Interacting Proteins: 2004 update.. Nucleic Acids Res.

[44] Suzuki R, Shimodaira H (2006). Pvclust: an R package for assessing the uncertainty in hierarchical clustering.. Bioinformatics.

