# Supplementary figures and images for: A Gene-Phenotype Network for the Laboratory Mouse and Its Implications for Systematic Phenotyping
Source: PLoS One. 2011 May 19;6(5):e19693. doi: 10.1371/journal.pone.0019693 (PMC3098258; doi:10.1371/journal.pone.0019693)

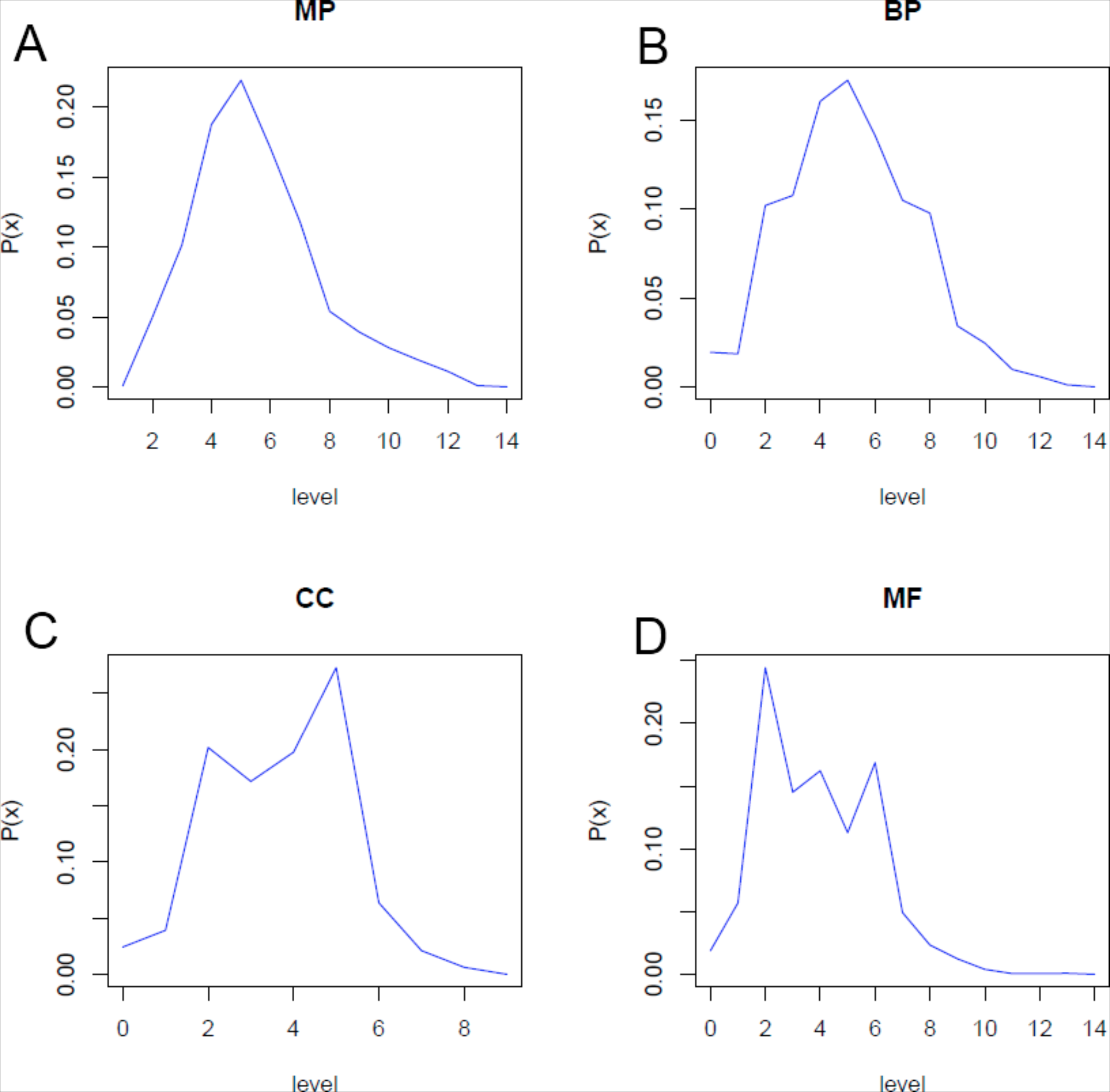

Supplement: Figure S1 — Probability mass for the frequency of annotations at different levels of the MP in the amalgamated list of gene-phenotype associations from MGI and Europhenome and the three GOs in Ensembl. (TIFF) [file pone.0019693.s001.tiff]

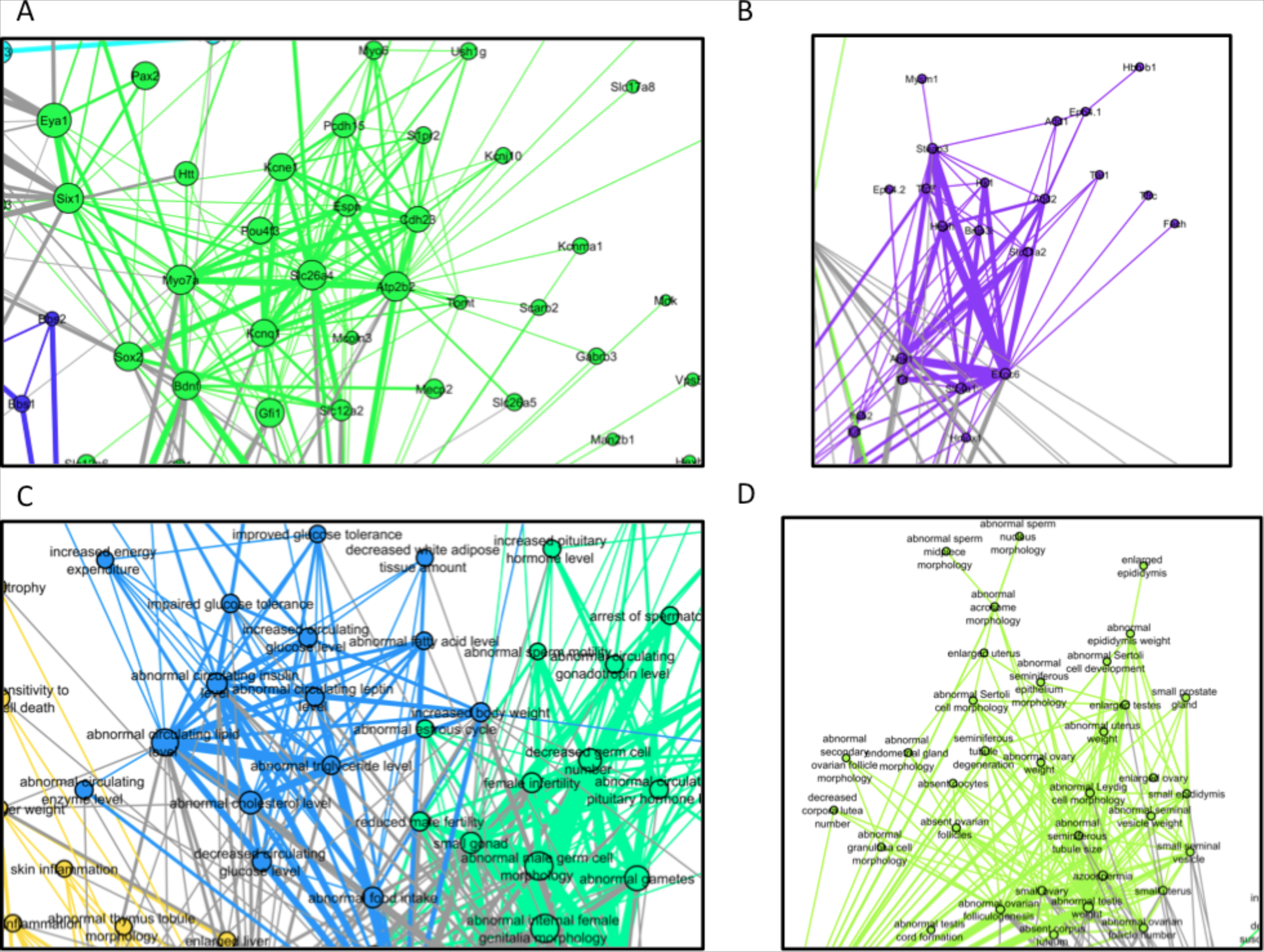

Supplement: Figure S2 — Details of example gene communities: A) Community 2 in the MP level 5 gene network. This community visually corresponds to the community that is frequently annotated with the granularity level 1 MP term “hearing/vestibular/ear phenotype” [MP:0005377] and is highly enriched in the GO terms “mechanoreceptor differentiation” [GO:0042490] and “sensory perception” [GO:0007600]. B) Community 5 in the MP level 8 gene network. This community visually corresponds to the community that is frequently annotated with the granularity level 1 MP term “hematopoietic system phenotype” [MP:0005397] and is highly enriched in the GO terms “erythrocyte homeostasis” [GO:0034101]. Details of example phenotype communities: C) Example of a phenotype community at level 5, illustrating a community of phenotypes related to diabetes (in blue). D) Example of a phenotype community at level 8 illustrating a community pf phenotypes related to reproductive system abnormalities (in green). (TIFF) [file pone.0019693.s002.tiff]

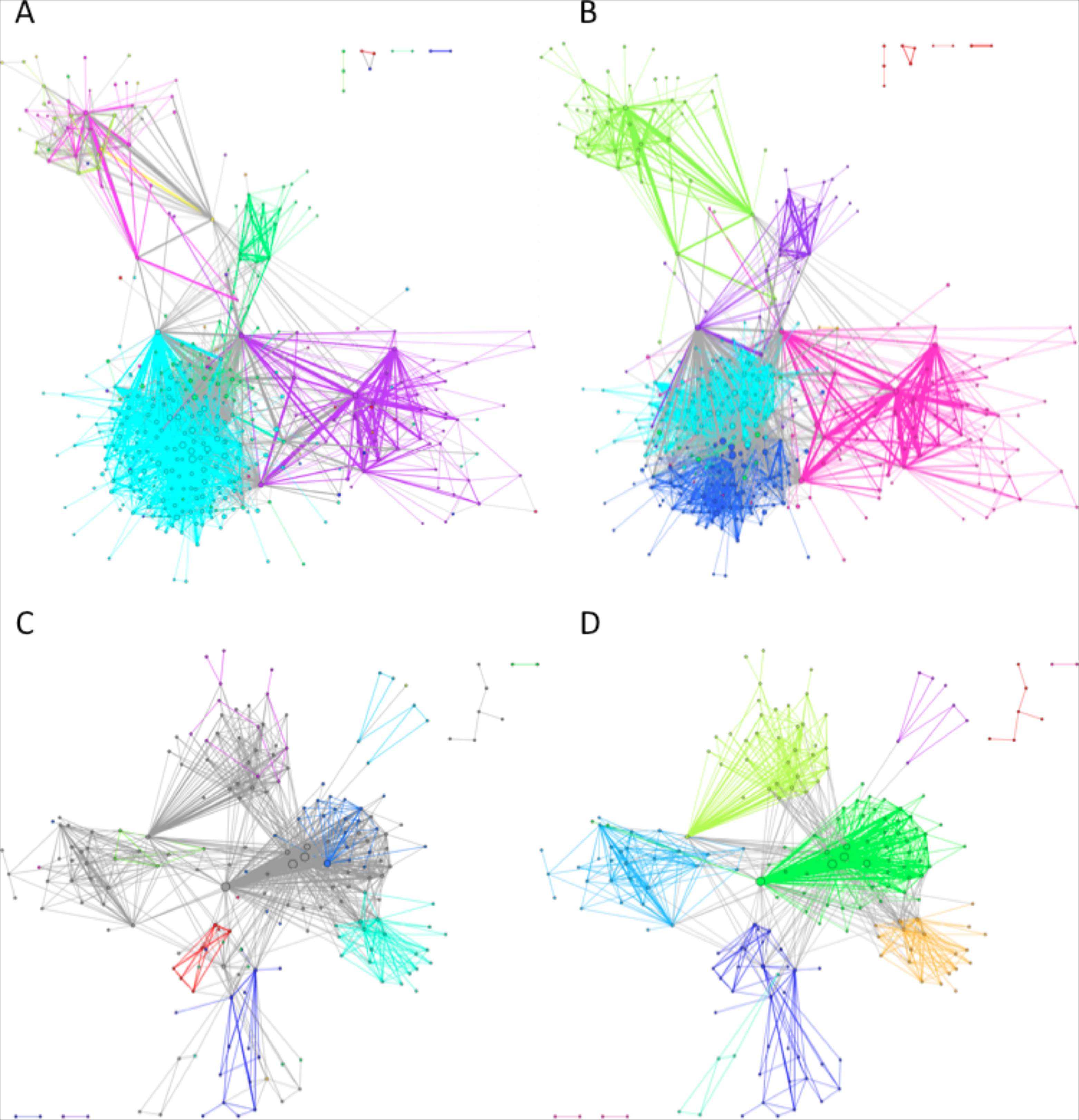

Supplement: Figure S3 — Gene and phenotype networks at MP level 8. Attributes are as in Fig. 1 A–D. (TIFF) [file pone.0019693.s003.tiff]

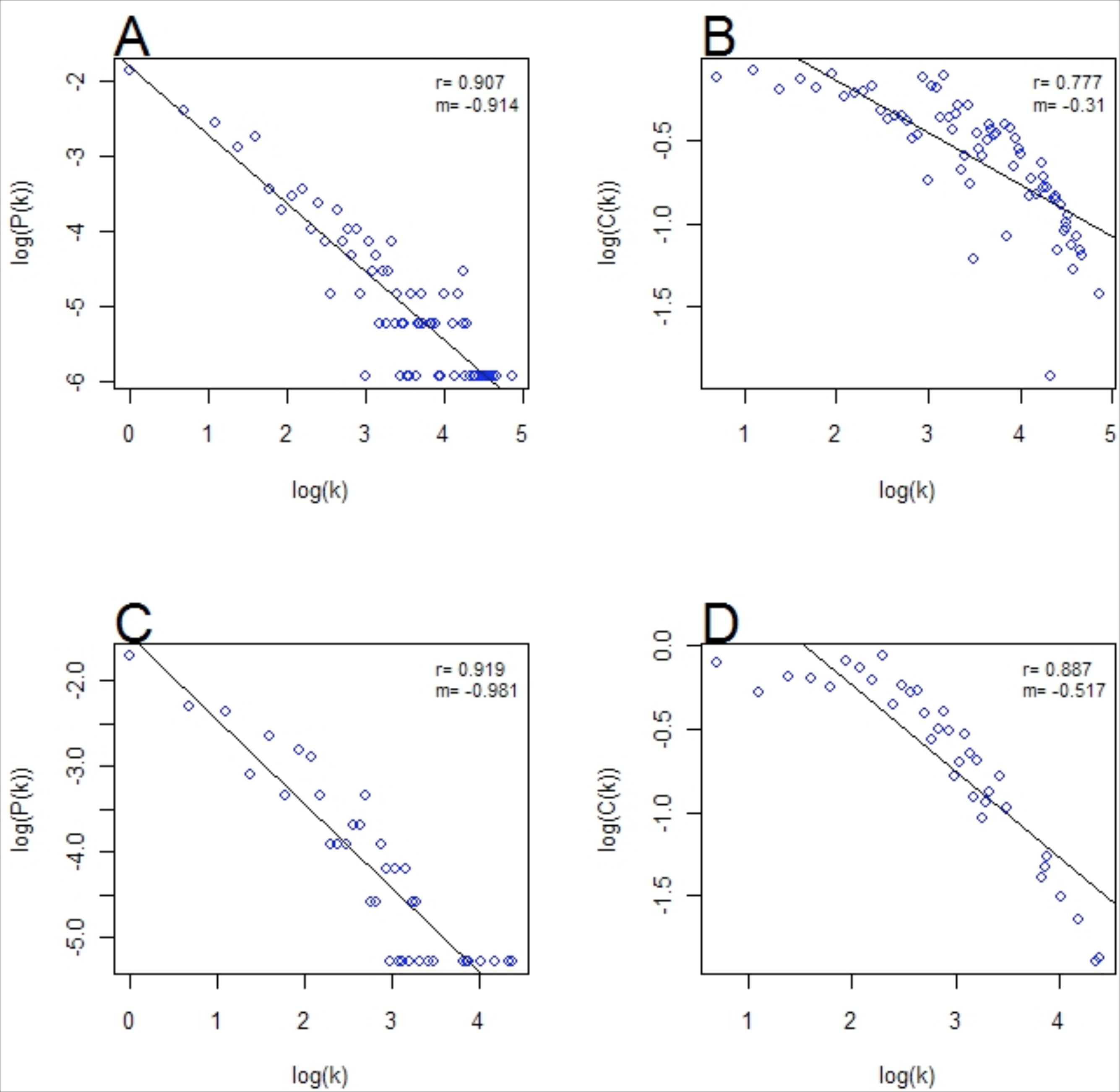

Supplement: Figure S4 — Topological analysis plots of level 8 networks, with cut-off. A)–B) Gene network degree distribution and clustering coefficient for the gene network at MP level 8, d cutoff = 0.011. C)–D) Phenotype Network degree distribution and the clustering coefficient distribution for the phenotype network at level 8, d cutoff = 0.002. Black lines indicate a line of best fit using linear regression. (TIFF) [file pone.0019693.s004.tiff]

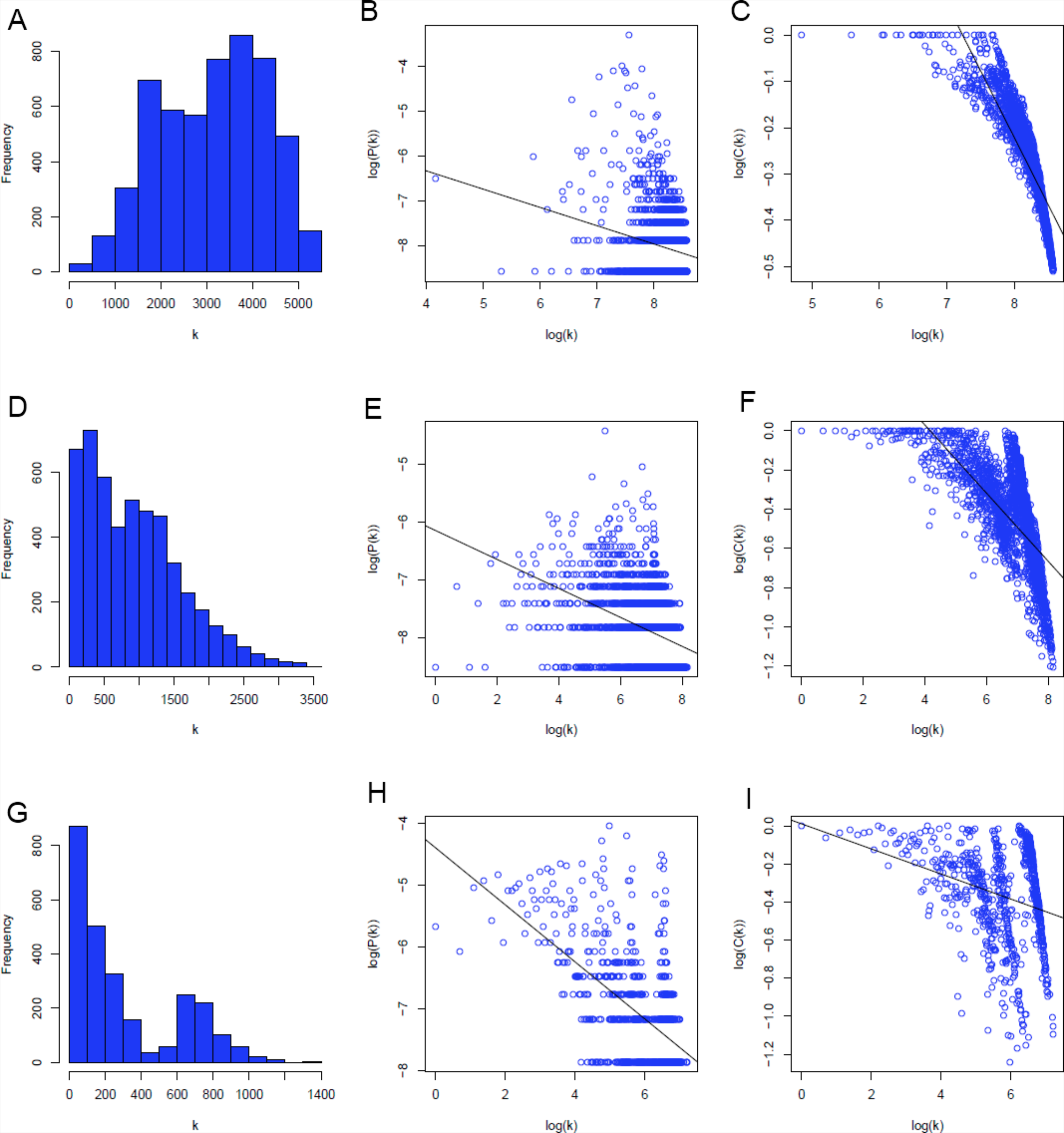

Supplement: Figure S5 — Topological analysis plots of level 5 gene networks, with no d cut-off. A–B) Gene network degree distribution and C) clustering coefficient distribution for the gene network at MP level 1, d cutoff = 0. D–F) The same quantities for MP level 5. G–I) The same quantities for MP level 8. Black lines indicate a line of best fit using linear regression. (TIFF) [file pone.0019693.s005.tiff]

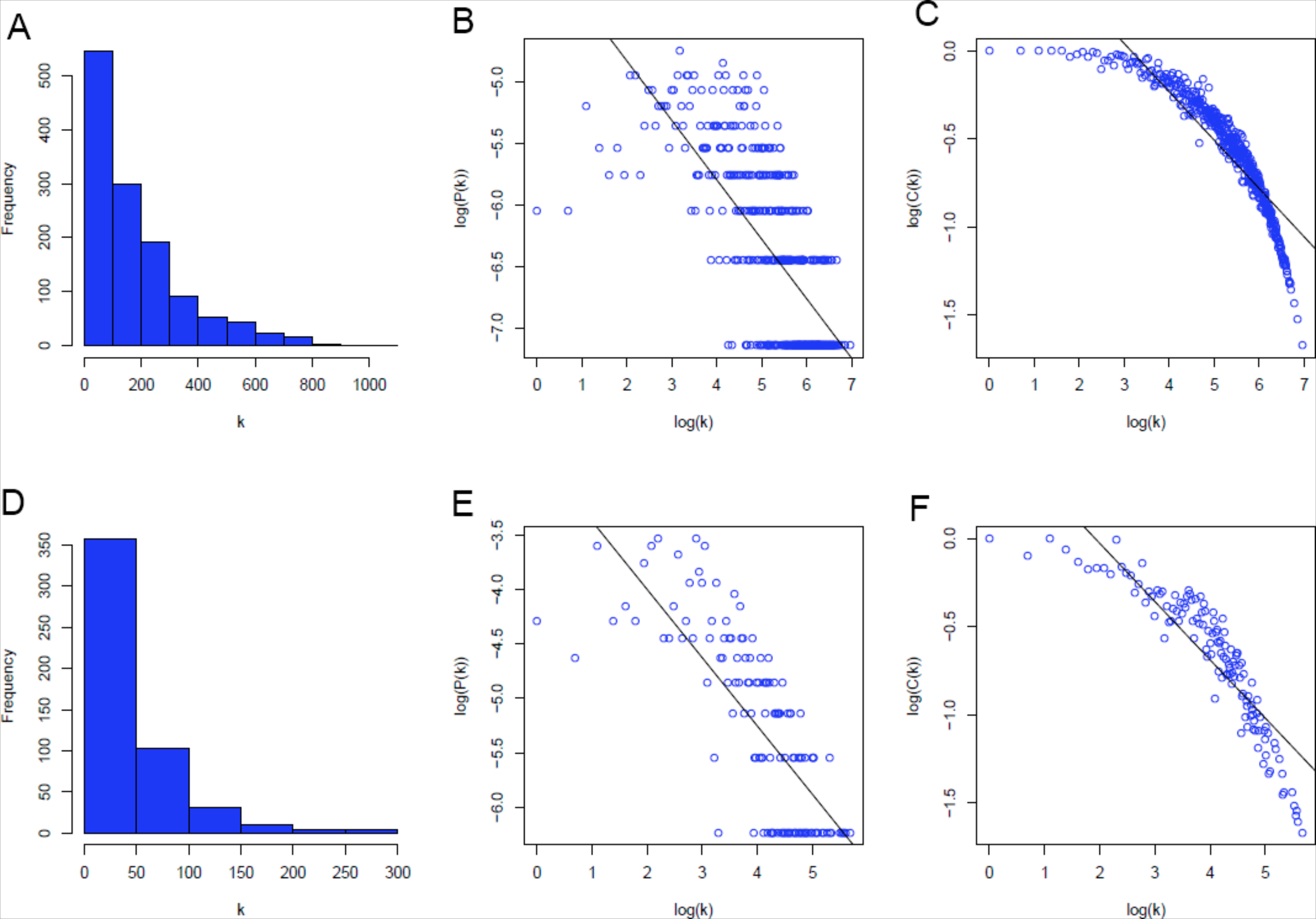

Supplement: Figure S6 — Topological analysis plots of phenotype networks with no cut-off. A–B) Gene network degree distribution and C) clustering coefficient distribution for the gene network at MP level 5, d cutoff = 0. D–F) The same quantities for MP level 8. (TIFF) [file pone.0019693.s006.tiff]

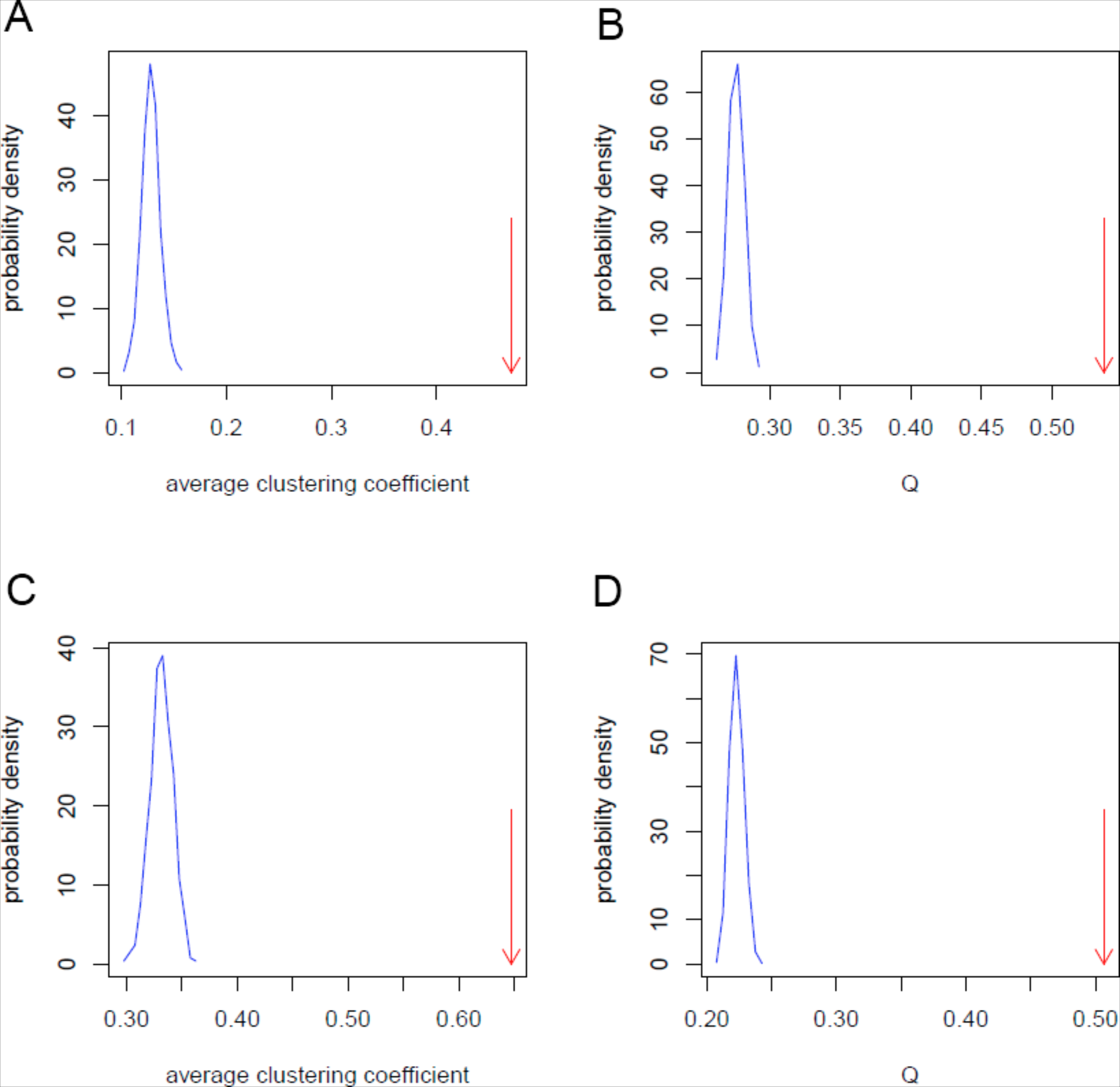

Supplement: Figure S7 — Modularity in the gene and phenotype networks. A)–B) Average clustering coefficient and Newman's modularity (Q) for randomly re-wired networks (blue) and the corresponding gene network at MP level 5 shown in Fig. 4.d cutoff = 0.011. The random networks retain the same degree distribution as the empirical gene network. C)–D) Average clustering coefficient and Newman's modularity (Q) for randomly re-wired networks (blue) and the corresponding phenotype network at MP level 5 shown in Fig. d cutoff = 0.005. (TIFF) [file pone.0019693.s007.tiff]

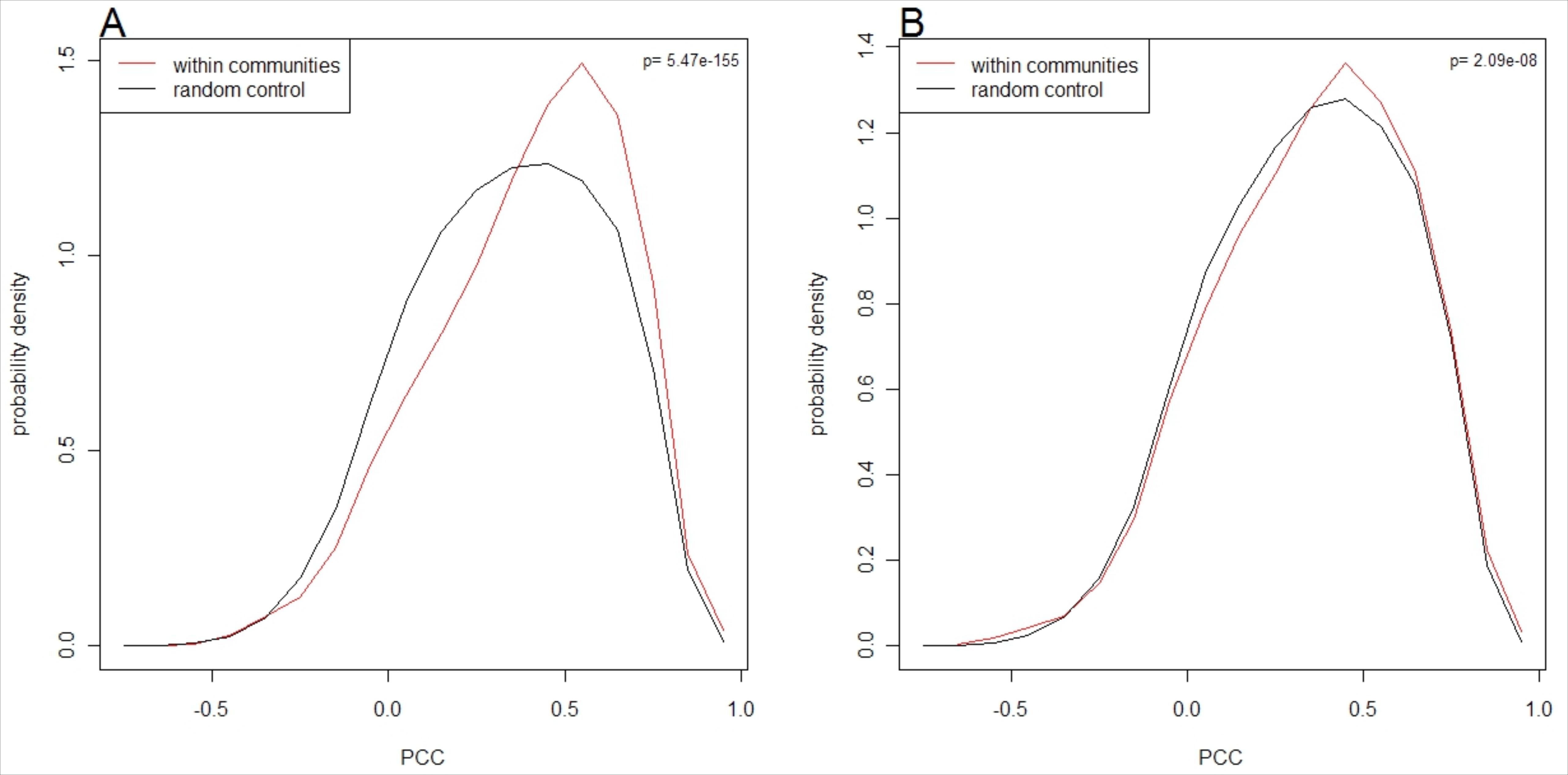

Supplement: Figure S8 — Phylogenetic profile correlation in communities. Shown are probability densities for genes within communities (red) compared to a random control (black) for the gene networks presented in the text at level 5 (A) and 8 (B). (TIFF) [file pone.0019693.s008.tiff]
