# Supplementary material for: A Gene-Phenotype Network for the Laboratory Mouse and Its Implications for Systematic Phenotyping
Source: PLoS One. 2011 May 19;6(5):e19693. doi: 10.1371/journal.pone.0019693 (PMC3098258; doi:10.1371/journal.pone.0019693)
Supplement: Table S1 — Top level MP terms that can form discrete communities, form communities which fall below the stringency cut-off (P<0.0001), or do not form communities. Communities are assessed within the phenotype networks constructed at MP levels 5 and 8, respectively. (PDF) [file pone.0019693.s011.pdf]

MP Level 5	MP Level 8
**Can be assigned to a discrete community**	
cardiovascular system phenotype	craniofacial phenotype
hearing/vestibular/ear phenotype	**hematopoietic system phenotype**
**hematopoietic system phenotype**	**immune system phenotype**
homeostasis/metabolism phenotype	**reproductive system phenotype**
**immune system phenotype**	**skeleton phenotype**
nervous system phenotype	
pigmentation phenotype	
renal/urinary system phenotype	
**reproductive system phenotype**	
**skeleton phenotype**	
tumorigenesis	
**Have genes in community but below cut-off**	
adipose tissue phenotype	**behavior/neurological phenotype**
**behavior/neurological phenotype**	cardiovascular system phenotype
cellular phenotype	**endocrine/exocrine gland phenotype**
craniofacial phenotype	hearing/vestibular/ear phenotype
digestive/alimentary phenotype	homeostasis/metabolism phenotype
embryogenesis phenotype	limbs/digits/tail phenotype
**endocrine/exocrine gland phenotype**	**muscle phenotype**
**muscle phenotype**	nervous system phenotype
**skin/coat/nails phenotype**	pigmentation phenotype
**vision/eye phenotype**	**skin/coat/nails phenotype**
	tumorigenesis
	**vision/eye phenotype**
**Not in network**	
**growth/size phenotype**	adipose tissue phenotype
**lethality-postnatal**	cellular phenotype
**lethality-prenatal/perinatal**	digestive/alimentary phenotype
**life span-post-weaning/aging**	embryogenesis phenotype
limbs/digits/tail phenotype	**growth/size phenotype**
**liver/biliary system phenotype**	**lethality-postnatal**
**other phenotype**	**lethality-prenatal/perinatal**
**respiratory system phenotype**	**life span-post-weaning/aging**
**taste/olfaction phenotype**	**liver/biliary system phenotype**
**touch/vibrissae phenotype**	**other phenotype**
	renal/urinary system phenotype
	**respiratory system phenotype**
	**taste/olfaction phenotype**
	**touch/vibrissae phenotype**

BOLD = present in same category at level 5 and level 8
